# Supplementary figures and images for: Brain-imaging evidence for compression of binary sound sequences in human memory
Source: eLife. 2023 Nov 1;12:e84376. doi: 10.7554/eLife.84376 (PMC10619979; doi:10.7554/eLife.84376)

**
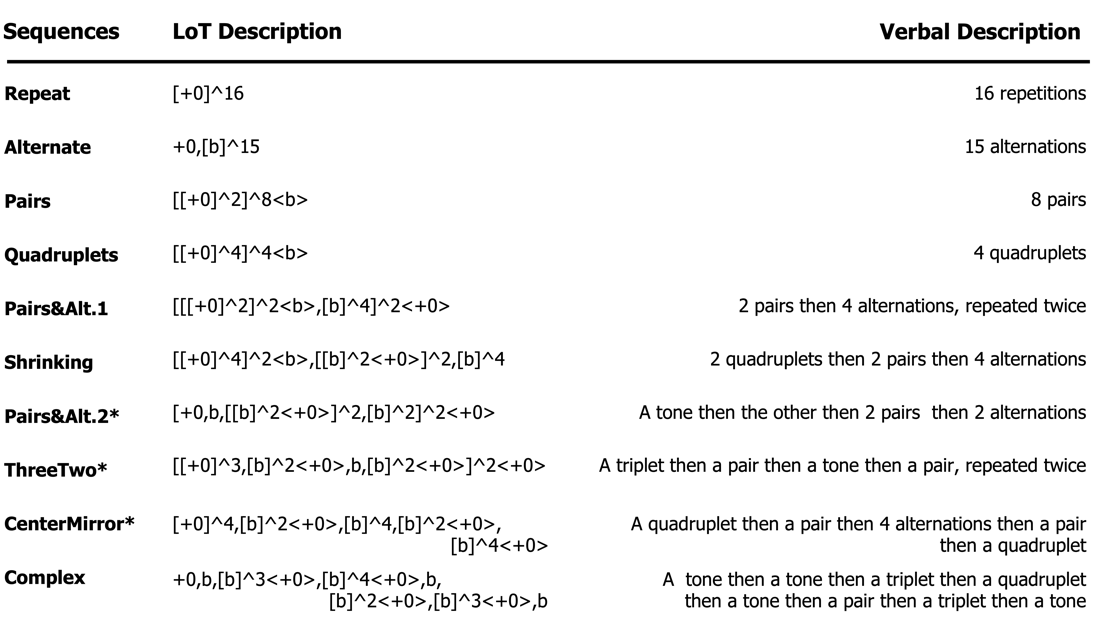
**

Supplement: Supplementary file 1. — The first column indicates the list of the different 16-item sequences used in the magneto-encephalography (MEG) and fMRI experiments. *Sequences used only in the fMRI experiment. The second column provides the sequence description obtained from the LoT. The third column is its verbal description, meant to ease the understanding of the formal expression. [file elife-84376-supp1.docx]
