# Supplementary material for: Brain-imaging evidence for compression of binary sound sequences in human memory
Source: eLife. 2023 Nov 1;12:e84376. doi: 10.7554/eLife.84376 (PMC10619979; doi:10.7554/eLife.84376)
Supplement: Supplementary file 2. [file elife-84376-supp2.docx]

| ***Positive LoT complexity effect in standard trials*** | |  |  |  |  |  |  |  |  |
| --- | --- | --- | --- | --- | --- | --- | --- | --- | --- |
|  | **Region** | **H** | **k** | **p(unc.)** | **p(FWE-corr)** | **T** | **x** | **y** | **z** |
|  | Superior parietal gyrus, Precuneus | L/R | 3254 | <.0001 | <.05 | 5.40 | -12 | -66 | 54 |
|  |  |  |  | <.0001 | 0.174 | 4.34 | -24 | -65 | 36 |
|  |  |  |  | <.0001 | 0.214 | 4.27 | -26 | -61 | 44 |
|  | Lobule VI of cerebellar hemisphere | R | 574 | <.0001 | <.05 | 5.09 | 27 | -60 | -26 |
|  | Precentral gyrus, Superior frontal gyrus (dorsolateral) | L | 1008 | <.0001 | <.05 | 4.88 | -36 | -4 | 54 |
|  |  |  |  | <.0001 | 0.177 | 4.34 | -27 | 0 | 70 |
|  |  |  |  | <0.001 | 0.956 | 3.43 | -15 | 2 | 63 |
|  | Lobule VIII of cerebellar hemisphere | L | 553 | <.0001 | 0.147 | 4.39 | -4 | -79 | -42 |
|  |  |  |  | <.0001 | 0.533 | 3.92 | -12 | -72 | -48 |
|  |  |  |  | <.0001 | 0.581 | 3.88 | -20 | -65 | -49 |
| ***Negative LoT complexity effect in standard trials*** | |  |  |  |  |  |  |  |  |
|  | **Region** | **H** | **k** | **p(unc.)** | **p(FWE-corr)** | **T** | **x** | **y** | **z** |
|  | Superior frontal gyrus (medial), Superior frontal gyrus (dorsolateral) | L/R | 6072 | <.0001 | <.05 | 5.29 | 13 | 61 | 33 |
|  |  |  |  | <.0001 | <.05 | 5.08 | 10 | 60 | 42 |
|  |  |  |  | <.0001 | <.05 | 5.05 | -26 | 60 | 28 |
|  | IFG pars orbitalis, Lateral orbital gyrus, Posterior orbital gyrus | L | 1093 | <.0001 | <.05 | 5.02 | -50 | 28 | -12 |
|  |  |  |  | <.0001 | 0.282 | 4.18 | -38 | 42 | -12 |
|  | Putamen | L | 713 | <.0001 | <.05 | 4.71 | -27 | -10 | 3 |
|  |  |  |  | <.0001 | 0.079 | 4.58 | -29 | -12 | -9 |
|  |  |  |  | <0.001 | 0.860 | 3.60 | -26 | -2 | -14 |
|  | Inferior occipital gyrus, Middle occipital gyrus | L | 561 | <.0001 | 0.051 | 4.70 | -26 | -98 | -9 |
|  | Inferior temporal gyrus, Middle temporal gyrus | R | 1035 | <.0001 | 0.068 | 4.62 | 50 | -7 | -30 |
|  |  |  |  | <.0001 | 0.477 | 3.97 | 52 | -23 | -18 |
|  |  |  |  | <0.001 | 0.912 | 3.53 | 64 | -19 | -21 |
|  | Angular gyrus, SupraMarginal gyrus | R | 463 | <.0001 | 0.084 | 4.56 | 64 | -51 | 31 |
|  |  |  |  | <0.001 | 0.995 | 3.23 | 52 | -63 | 44 |
|  | Putamen | R | 543 | <.0001 | 0.094 | 4.53 | 30 | -7 | 2 |
|  | Middle cingulate & paracingulate gyri | L/R | 1434 | <.0001 | 0.129 | 4.43 | 6 | -23 | 42 |
|  |  |  |  | <.0001 | 0.269 | 4.20 | -4 | -23 | 40 |
|  |  |  |  | <0.001 | 0.690 | 3.78 | 13 | -49 | 36 |
|  | Angular gyrus, Inferior parietal gyrus | L | 1120 | <.0001 | 0.171 | 4.35 | -55 | -58 | 31 |
|  |  |  |  | <.0001 | 0.466 | 3.98 | -60 | -51 | 42 |
|  | Inferior temporal gyrus | L | 614 | <.0001 | 0.380 | 4.07 | -54 | -9 | -37 |
|  |  |  |  | <0.001 | 0.844 | 3.62 | -46 | -19 | -23 |
|  |  |  |  | <0.001 | 0.983 | 3.33 | -54 | -24 | -18 |
