# Supplementary material for: Brain-imaging evidence for compression of binary sound sequences in human memory
Source: eLife. 2023 Nov 1;12:e84376. doi: 10.7554/eLife.84376 (PMC10619979; doi:10.7554/eLife.84376)
Supplement: Supplementary file 3. [file elife-84376-supp3.docx]

| ***Positive LoT complexity effect in deviant trials*** | |  |  |  |  |  |  |  |  |
| --- | --- | --- | --- | --- | --- | --- | --- | --- | --- |
|  | **Region** | **H** | **k** | **p(unc.)** | **p(FWE-corr)** | **T** | **x** | **y** | **z** |
|  | Superior frontal gyrus (medial) | L/R | 474 | <.0001 | 0.218 | 4.33 | -1 | 60 | 38 |
|  |  |  |  | <0.001 | 0.773 | 3.77 | -6 | 49 | 54 |
|  |  |  |  | <0.001 | 0.999 | 3.20 | 3 | 46 | 42 |
| ***Negative LoT complexity effect in deviant trials*** | |  |  |  |  |  |  |  |  |
|  | **Region** | **H** | **k** | **p(unc.)** | **p(FWE-corr)** | **T** | **x** | **y** | **z** |
|  | Superior and middle temporal, Precentral, Postcentral, SupraMarginal, Inferior parietal gyri, Insula, Rolandic operculum, Cerebellum (Lobule VI), Putamen | L/R | 46888 | <.0001 | <.0001 | 7.58 | -57 | -21 | 26 |
|  |  |  |  | <.0001 | <.0001 | 7.23 | -36 | 0 | 5 |
|  |  |  |  | <.0001 | <.0001 | 6.97 | -48 | -60 | 5 |
|  | Middle cingulate & paracingulate gyri, Supplementary motor area | L/R | 5241 | <.0001 | <.0001 | 7.12 | -3 | -5 | 54 |
|  |  |  |  | <.0001 | <.0001 | 6.74 | -6 | 11 | 37 |
|  |  |  |  | <.0001 | <.05 | 4.87 | -10 | -26 | 44 |
|  | Lobule VIII of cerebellar hemisphere | R | 1242 | <.0001 | <0.001 | 5.94 | 18 | -63 | -51 |
|  |  |  |  | <.0001 | 0.508 | 4.01 | 17 | -79 | -53 |
|  | Lobule VIII of cerebellar hemisphere | L | 848 | <.0001 | 0.051 | 4.75 | -24 | -58 | -51 |
|  |  |  |  | <.0001 | 0.066 | 4.68 | -17 | -63 | -55 |
|  |  |  |  | <0.001 | 0.999 | 3.19 | -3 | -63 | -44 |
|  | Calcarine fissure, Lingual gyrus | L/R | 764 | <.0001 | 0.137 | 4.47 | 11 | -70 | 5 |
|  |  |  |  | <0.001 | 0.887 | 3.64 | -13 | -75 | 5 |
|  |  |  |  | <0.001 | 0.945 | 3.54 | -20 | -67 | 5 |
| ***Positive LoT complexity effect in correctly-detected deviant trials*** | | |  |  |  |  |  |  |  |
|  | **Region** | **H** | **k** | **p(unc.)** | **p(FWE-corr)** | **T** | **x** | **y** | **z** |
|  | Superior frontal gyrus (medial) | L/R | 1584 | <.0001 | <0.001 | 5.79 | -1 | 37 | 44 |
|  |  |  |  | <.0001 | 0.225 | 4.30 | -3 | 23 | 58 |
|  |  |  |  | <0.001 | 0.944 | 3.52 | 10 | 42 | 23 |
| ***Negative LoT complexity effect in correctly-detected deviant trials*** | | |  |  |  |  |  |  |  |
|  | **Region** | **H** | **k** | **p(unc.)** | **p(FWE-corr)** | **T** | **x** | **y** | **z** |
|  | Superior temporal gyrus, Insula, Temporal pole | R | 2359 | <.0001 | <.05 | 5.54 | 41 | -5 | -11 |
|  |  |  |  | <.0001 | <.05 | 5.19 | 52 | 0 | -7 |
|  |  |  |  | <.0001 | 0.397 | 4.10 | 62 | -11 | 5 |
|  | Superior temporal gyrus, Insula | L | 1694 | <.0001 | <.05 | 5.02 | -48 | -4 | -9 |
|  |  |  |  | <.0001 | 0.075 | 4.63 | -33 | -5 | -4 |
|  |  |  |  | <.0001 | 0.206 | 4.33 | -52 | -5 | 9 |
|  | Middle temporal gyrus, Superior temporal gyrus | R | 1692 | <.0001 | <.05 | 4.76 | 60 | -47 | 9 |
|  |  |  |  | <.0001 | 0.174 | 4.38 | 55 | -30 | 3 |
|  |  |  |  | <.0001 | 0.234 | 4.29 | 57 | -58 | 3 |
|  | SupraMarginal gyrus, Middle temporal gyrus | L | 1727 | <.0001 | 0.110 | 4.52 | -48 | -54 | -2 |
|  |  |  |  | <.0001 | 0.112 | 4.52 | -54 | -37 | 28 |
|  |  |  |  | <.0001 | 0.239 | 4.28 | -59 | -67 | 10 |
|  | Supplementary motor area, Middle cingulate & paracingulate gyri | L/R | 807 | <.0001 | 0.127 | 4.48 | 1 | -7 | 56 |
|  |  |  |  | <.0001 | 0.232 | 4.29 | 3 | 0 | 44 |
